# Supplementary material for: Functional outcomes and quality of life after a 6-month early intervention program for oral cancer survivors: a single-arm clinical trial
Source: PeerJ. 2018 Feb 21;6:e4419. doi: 10.7717/peerj.4419 (PMC5827017; doi:10.7717/peerj.4419)
Supplement: Supplemental Information 4 — The demographic comparison between the drop-out and the analytic groups. [file peerj-06-4419-s004.docx]

**Table sup.** Demographic and clinical characteristics of the total participants.

|  |  | **Drop-out** | **Analyzed** | **p-value** |  |
| --- | --- | --- | --- | --- | --- |
| Age | | 40.7 ± 10.1 | 51.3 ± 10.3 | .229 |  |
| Gender | F | 1 (3%) | 5 (5%) | .660 |  |
|  | M | 32 (97%) | 60 (95%) |  |  |
| Education | < 9 years | 18 (56%) | 26 (41%) | .369 |  |
|  | 9-12 years | 12 (38%) | 29 (45%) |  |  |
|  | > 12years | 2(6%) | 9 (14%) |  |  |
| Marriage | Single | 6 (19%) | 7 (11%) | .034 |  |
|  | Married | 17 (53%) | 51 (79%) |  |  |
|  | Other | 9 (28%) | 7 (11%) |  |  |
| Breadwinner | N | 12 (37%) | 20 (31%) | .647 |  |
|  | Y | 20 (63%) | 44 (69%) |  |  |
| Vocation | Retired/unemployed | 2 (7%) | 8 (12%) | .261 |  |
|  | Self-employed | 2 (7%) | 15 (23%) |  |  |
|  | Professional | 5 (19%) | 5 (8%) |  |  |
|  | Administration | 3 (11%) | 3 (5%) |  |  |
|  | Service | 7 (26%) | 10 (15%) |  |  |
|  | Semi-skilled/skilled  skilled worker | 8 (30%) | 24 (37%) |  |  |
| ND | Right | 11 (33%) | 25 (39%) | .673 |  |
|  | Left | 16 (49%) | 25 (39%) |  |  |
|  | Bilateral | 6 (18%) | 15 (23%) |  |  |
| Area of tumor | Buccal | 12 (36%) | 21 (32%) | .949 |  |
|  | Tongue | 6 (15%) | 18 (28%) |  |  |
|  | Mouth floor | 0 (0%) | 7 (11%) |  |  |
|  | Gum | 5 (15%) | 8 (12%) |  |  |
|  | Pharyngeal wall | 0 (0%) | 2 (3%) |  |  |
|  | Lip | 2 (6%) | 3 (5%) |  |  |
|  | Retromolar tumor | 7 (21%) | 4 (6%) |  |  |
|  | Gingival | 1 (3%) | 1 (2%) |  |  |
|  | Hard palate | 1 (3%) | 1 (2%) |  |  |
|  | Soft palate | 0 (0%) | 1 (2%) |  |  |
| T stage | 1 | 4 (12%) | 8 (12%) | .828 |  |
|  | 2 | 14 (42%) | 30 (46%) |  |  |
|  | 3 | 2 (6%) | 7 (11%) |  |  |
|  | 4 | 13 (39%) | 20 (31%) |  |  |
| N stage | 0 | 17 (52%) | 37(57%) | .501 |  |
|  | 1 | 2 (6%) | 8 (12%) |  |  |
|  | 2 | 14 (42%) | 19 (29%) |  |  |
|  | 3 | 0 (0%) | 1 (2%) |  |  |
| M stage | 0 | 33 (100%) | 65 (100%) |  |  |
| Radiation therapy* | No | 18 (62%) | 28 (44%) | .121 |  |
|  | Yes | 11 (38%) | 36 (56%) |  |  |
| Donor site | Anterolateral thigh flap | 21 (64%) | 40 (62%) | .539 |  |
|  | Fibular osteoseptocutaneous flap | 4 (12%) | 10 (15%) |  |  |
|  | Other | 8 (24%) | 25 (23%) |  |  |
