# Supplementary material for: Functional outcomes and quality of life after a 6-month early intervention program for oral cancer survivors: a single-arm clinical trial
Source: PeerJ. 2018 Feb 21;6:e4419. doi: 10.7717/peerj.4419 (PMC5827017; doi:10.7717/peerj.4419)
Supplement: Supplemental Information 5 [file peerj-06-4419-s005.pdf]

ClinicalTrials.gov PRS DRAFT Receipt (Working Version)
Last Update: 06/22/2017 10:47

ClinicalTrials.gov ID: NCT03206242

Study Identification

Unique Protocol ID: 104-2300C

Brief Title: Rehabilitation Outcomes in Head and Neck Survivors (HNC)

Official Title: Rehabilitation Outcomes in Head and Neck Survivors

Secondary IDs: CGMH-IRB-103-5164B [Registry ID: CGMH]

Study Status

Record Verification: May 2015

Overall Status: Completed

Study Start: January 1, 2015 [Actual]

Primary Completion: June 30, 2016 [Actual]

Study Completion: June 30, 2016 [Actual]

Sponsor/Collaborators

Sponsor: Chang Gung Memorial Hospital

Responsible Party: Sponsor

Collaborators:

Oversight

U.S. FDA-regulated Drug:

U.S. FDA-regulated Device:

U.S. FDA IND/IDE: No

Human Subjects Review: Board Status: Approved
Approval Number: 103-5164B
Board Name: Institutional Review Board
Board Affiliation: Chang Gung Memorial Hospital
Phone: +886-3-3196200
Email: tsengshui@cgmh.org.tw
Address:

B2., No.123, Dinghu Rd., Gueishan Township,
Taoyuan County 333, Taiwan

Data Monitoring: Yes

FDA Regulated Intervention: No

Study Description

Brief Summary: Head and neck cancer is prevalent in Taiwan, and oral cancer is the most common location. Patients with advanced stage of the disease need extensive tumor excision with neck dissection. Secondary reconstructive surgeries using free flap could improve the postoperative function or appearance of cancer survivors. Advanced treatments make survival rates increased. Effects of treatment for oral cancer develop shoulder dysfunction, speech, mastication, donor site morbidity and psychological issues. Physical therapy may have benefits for temporomandibular joint function, shoulder pain relief, muscle performance, and oral structures coordination. Return to work in the number of cancer survivors is a realistic outcome. Rehabilitation effects on functional restorations and quality of life for head and neck survivors are needed for further studied.

The purpose of this project is to explore the rehabilitation effects following head and neck reconstructive survivors. The investigators measure temporomandibular joint function, shoulder function, pain monthly. Physical functions, self-reported quality of life, and the status of return to work are measured 3 and 6 months after surgery. This prospective study could help to predict the rehabilitation outcomes and benefits.

Detailed Description: Head and neck cancer is prevalent in Taiwan, and oral cancer is the most common location. Patients with advanced stage of the disease need extensive tumor excision with neck dissection. Secondary reconstructive surgeries using free flap could improve the postoperative function or appearance of cancer survivors. Oral functions include respiration, speech, mastication, deglutition, and cosmetics. Advanced treatments make survival rates increased, but might develop shoulder dysfunction, speech, mastication, donor site morbidity and psychological issues. The purpose of this project is to explore the effects of rehabilitation following reconstructive surgery in oral cancer survivors.

This study design is an interrupted time-series design. The investigators will recruit 50 subjects one week following reconstructive surgery. The measurements include manual muscle strength, joint range of motion, maximal mouth opening, pain status, hand-to-neck test, hand-to-scapula test, hand-to-opposite-scapula test, 6-minute walking test, timed up & go test, European Organization for Research and Treatment of Cancer (EORTC) quality of life questionnaire (QLQ)-C30, European Organization for Research and Treatment of Cancer (EORTC) quality of life questionnaire (QLQ)-H&N35 and return-to-work. These tests were done at the first visit (0 week post-operation), three months and six months after reconstructive surgery respectively. The intervention programs consist of edema control, scar management, pain management, respiration training, oral function training, neck and shoulder function training, donor site mobility training. Continuous variables were analyzed by descriptive statistics. One-way ANOVA was used to compare the difference between measurements. Binary logistic regression was used to predict the factors of return-to-work.

Conditions

Conditions: Head and Neck Cancer
Oral Cancer

Keywords: Rehabilitation
Physiotherapy
Trismus
Pain
Return to work

Quality of life

Study Design

Study Type: Observational

Observational Study Model: Cohort

Time Perspective: Prospective

Biospecimen Retention: None Retained

Biospecimen Description:

Enrollment: 65 [Actual]

Number of Groups/Cohorts: 3

Groups and Interventions

Groups/Cohorts	Interventions
initial<br>0 month begin physiotherapy	Physiotherapy<br>Interventions of physical therapy will be implemented after reconstructive surgery including edema control, scar management, pain management, respiration training, oral function training, neck and shoulder function training, donor site mobility training.<br>Other Names:<br>• Rehabilitation
3 months after physiotherapy<br>3 months after physiotherapy	Physiotherapy<br>Interventions of physical therapy will be implemented after reconstructive surgery including edema control, scar management, pain management, respiration training, oral function training, neck and shoulder function training, donor site mobility training.<br>Other Names:<br>• Rehabilitation
6 months after physiotherapy<br>6 months after physiotherapy	Physiotherapy<br>Interventions of physical therapy will be implemented after reconstructive surgery including edema control, scar management, pain management, respiration training, oral function training, neck and shoulder function training, donor site mobility training.<br>Other Names:<br>• Rehabilitation

Outcome Measures

Primary Outcome Measure:

1. return to work as measured by interview
   [Time Frame: 1 year]
2. quality of life as measured by European Organization for Research and Treatment of Cancer (EORTC) quality of life questionnaire (QLQ)-C30
   [Time Frame: 1 year]
3. physical functions measured by 6-minute walking test
   [Time Frame: 1 year]
4. quality of life as measured by European Organization for Research and Treatment of Cancer (EORTC) quality of life questionnaire (QLQ)-H&N35
   [Time Frame: 1 year]
5. physical functions measured by time up & go test
   [Time Frame: 1 year]

Secondary Outcome Measure:

6. shoulder function measured by function-related tests
   [Time Frame: 1 year]
7. pain measured by Visual Analog Scale
   [Time Frame: 1 year]
8. mouth opening measured by Boley gauge
   [Time Frame: 1 year]
9. joint range of motion measured by goniometer
   [Time Frame: 1 year]
10. muscle strength measured by manual muscle testing
   [Time Frame: 1 year]

NOTE : Outcome Measure Description has not been entered.
NOTE : Outcome Measure Description has not been entered.
NOTE : Outcome Measure Description has not been entered.
NOTE : Outcome Measure Description has not been entered.
NOTE : Outcome Measure Description has not been entered.
NOTE : Outcome Measure Description has not been entered.
NOTE : Outcome Measure Description has not been entered.
NOTE : Outcome Measure Description has not been entered.
NOTE : Outcome Measure Description has not been entered.
NOTE : Outcome Measure Description has not been entered.
NOTE : Normally only one Primary Outcome Measure is specified.

Eligibility

Study Population: Subjects receive physical therapy at Linkou Chang Gung Memorial Hospital will be invited to participate this study.

Sampling Method: Non-Probability Sample

Minimum Age: 20 Years

Maximum Age: 65 Years

Sex: All

Gender Based:

Accepts Healthy Volunteers: No

Criteria: Inclusion Criteria:

- Clinical diagnosis of oral cancer
- Post-reconstructive surgery
- Age between 20 to 65 years old
- Must be able to follow instructions

Exclusion Criteria:

- Central nervous disease
- Metastasis

Contacts/Locations

Central Contact Person: Yueh-Hsia Chen, Master
Telephone: +886-3-3281200 Ext. 2170
Email: yuehhsia@cgmh.org.tw

Central Contact Backup: Yuan-Hung Chao, PhD
Telephone: +886-2-33668129
Email: yuanhungchao@ntu.edu.tw

Study Officials: Yueh-Hsia Chen, Master
Study Principal Investigator
Chang Gung Memorial Hospital

Locations: Taiwan
Department of Plastic and Reconstructive Surgery Rehabilitation Center, Chung Gung Memorial Hospital
Taoyuan, Taiwan, 333
Contact: Yueh-Hsia Chen, Master +886-3-3281200 Ext. 2170 yuehhsia@cgmh.org.tw
Contact: Yuan-Hung Chao, PhD +886-2-33668129 yuanhungchao@ntu.edu.tw

IPDSharing

Plan to Share IPD:

References

Citations:

[Study Results] Wong CH, Wei FC. Microsurgical free flap in head and neck reconstruction. Head Neck. 2010 Sep;32(9):1236-45. doi: 10.1002/hed.21284. Review. PubMed 20014446

[Study Results] Lutz BS, Wei FC. Microsurgical workhorse flaps in head and neck reconstruction. Clin Plast Surg. 2005 Jul;32(3):421-30, vii. Review. PubMed 15979480

[Study Results] Neligan PC. Head and neck reconstruction. Plast Reconstr Surg. 2013 Feb;131(2):260e-269e. doi: 10.1097/PRS.0b013e3182778938. PubMed 23358022

[Study Results] Pauloski BR, Rademaker AW, Logemann JA, Discekici-Harris M, Mittal BB. Comparison of swallowing function after intensity-modulated radiation therapy and conventional radiotherapy for head and neck cancer. Head Neck. 2015 Nov;37(11):1575-82. doi: 10.1002/hed.23796. Epub 2014 Aug 1. PubMed 24909649

[Study Results] Sheikh A, Shallwani H, Ghaffar S. Postoperative shoulder function after different types of neck dissection in head and neck cancer. Ear Nose Throat J. 2014 Apr-May;93(4-5):E21-6. PubMed 24817237

[Study Results] van der Molen L, van Rossum MA, Burkhead LM, Smeele LE, Rasch CR, Hilgers FJ. A randomized preventive rehabilitation trial in advanced head and neck cancer patients treated with chemoradiotherapy: feasibility, compliance, and short-term effects. Dysphagia. 2011 Jun;26(2):155-70. doi: 10.1007/s00455-010-9288-y. Epub 2010 Jul 11. PubMed 20623305

[Study Results] Eades M, Murphy J, Carney S, Amdouni S, Lemoignan J, Jelowicki M, Nadler M, Chasen M, Gagnon B. Effect of an interdisciplinary rehabilitation program on quality of life in patients with head and neck cancer: review of clinical experience. Head Neck. 2013 Mar;35(3):343-9. doi: 10.1002/hed.22972. Epub 2012 Mar 16. PubMed 22422558

[Study Results] McGarvey AC, Osmotherly PG, Hoffman GR, Chiarelli PE. Impact of neck dissection on scapular muscle function: a case-controlled electromyographic study. Arch Phys Med Rehabil. 2013 Jan;94(1):113-9. doi: 10.1016/j.apmr.2012.07.017. Epub 2012 Aug 1. PubMed 22864015

[Study Results] Hsieh LC, Chen JW, Wang LY, Tsang YM, Shueng PW, Liao LJ, Lo WC, Lin YC, Tseng CF, Kuo YS, Jhuang JY, Tien HJ, Juan HF, Hsieh CH. Predicting the severity and prognosis of trismus after intensity-modulated radiation therapy for oral cancer patients by magnetic resonance imaging. PLoS One. 2014 Mar 21;9(3):e92561. doi: 10.1371/journal.pone.0092561. eCollection 2014. PubMed 24658376

[Study Results] Wetzels JW, Merkx MA, de Haan AF, Koole R, Speksnijder CM. Maximum mouth opening and trismus in 143 patients treated for oral cancer: a 1-year prospective study. Head Neck. 2014 Dec;36(12):1754-62. doi: 10.1002/hed.23534. Epub 2014 Jan 30. PubMed 24478217

[Study Results] Lee R, Slevin N, Musgrove B, Swindell R, Molassiotis A. Prediction of post-treatment trismus in head and neck cancer patients. Br J Oral Maxillofac Surg. 2012 Jun;50(4):328-32. doi: 10.1016/j.bjoms.2011.06.009. Epub 2011 Jul 26. PubMed 21794962

[Study Results] Stubblefield MD. Radiation fibrosis syndrome: neuromuscular and musculoskeletal complications in cancer survivors. PM R. 2011 Nov;3(11):1041-54. doi: 10.1016/j.pmrj.2011.08.535. Review. PubMed 22108231

[Study Results] Chan JY, Lua LL, Starmer HH, Sun DQ, Rosenblatt ES, Gourin CG. The relationship between depressive symptoms and initial quality of life and function in head and neck cancer. Laryngoscope. 2011 Jun;121(6):1212-8. doi: 10.1002/lary.21788. Epub 2011 May 3. PubMed 21541945

[Study Results] EWING MR, MARTIN H. Disability following radical neck dissection; an assessment based on the postoperative evaluation of 100 patients. Cancer. 1952 Sep;5(5):873-83. PubMed 12988177

[Study Results] McNeely ML, Parliament MB, Seikaly H, Jha N, Magee DJ, Haykowsky MJ, Courneya KS. Effect of exercise on upper extremity pain and dysfunction in head and neck cancer survivors: a randomized controlled trial. Cancer. 2008 Jul 1;113(1):214-22. doi: 10.1002/cncr.23536. PubMed 18457329

[Study Results] Pauli N, Andréll P, Johansson M, Fagerberg-Mohlin B, Finizia C. Treating trismus: A prospective study on effect and compliance to jaw exercise therapy in head and neck cancer. Head Neck. 2015 Dec;37(12):1738-44. doi: 10.1002/hed.23818. Epub 2014 Sep 25. PubMed 24986051

[Study Results] Høgdal N, Juhl C, Aadahl M, Gluud C. Early preventive exercises versus usual care does not seem to reduce trismus in patients treated with radiotherapy for cancer in the oral cavity or oropharynx: a randomised clinical trial. Acta Oncol. 2015 Jan;54(1):80-7. doi: 10.3109/0284186X.2014.954677. Epub 2014 Sep 17. PubMed 25229260

[Study Results] Shepherd KL, Fisher SE. Prospective evaluation of quality of life in patients with oral and oropharyngeal cancer: from diagnosis to three months post-treatment. Oral Oncol. 2004 Aug;40(7):751-7. PubMed 15172646

[Study Results] Schliephake H, Jamil MU. Prospective evaluation of quality of life after oncologic surgery for oral cancer. Int J Oral Maxillofac Surg. 2002 Aug;31(4):427-33. PubMed 12361079

[Study Results] Hidalgo DA. Fibula free flap: a new method of mandible reconstruction. Plast Reconstr Surg. 1989 Jul;84(1):71-9. PubMed 2734406

[Study Results] Ling XF, Peng X. What is the price to pay for a free fibula flap? A systematic review of donor-site morbidity following free fibula flap surgery. Plast Reconstr Surg. 2012 Mar;129(3):657-74. doi: 10.1097/PRS.0b013e3182402d9a. Review. PubMed 22090247

[Study Results] Chou SW, Liao HT, Yazar S, Lin CH, Lin YC, Wei FC. Assessment of fibula osteoseptocutaneous flap donor-site morbidity using balance and gait test. J Orthop Res. 2009 Apr;27(4):555-60. doi: 10.1002/jor.20687. PubMed 18985681

[Study Results] Bartaire E, Mouawad F, Mallet Y, Milet P, El Bedoui S, Ton Van J, Chevalier D, Lefebvre JL. Morphologic assessment of mandibular reconstruction by free fibula flap and donor-site functional impairment in a series of 23 patients. Eur Ann Otorhinolaryngol Head Neck Dis. 2012 Oct;129(5):230-7. doi: 10.1016/j.anorl.2011.06.004. Epub 2012 Oct 15. PubMed 23078983

[Study Results] Cooper AF, Hankins M, Rixon L, Eaton E, Grunfeld EA. Distinct work-related, clinical and psychological factors predict return to work following treatment in four different cancer types. Psychooncology. 2013 Mar;22(3):659-67. doi: 10.1002/pon.3049. Epub 2012 Mar 21. PubMed 22434715

[Study Results] Ross L, Petersen MA, Johnsen AT, Lundstroem LH, Carlsen K, Groenvold M. Factors associated with Danish cancer patients' return to work. A report from the population-based study 'The Cancer Patient's World'. Cancer Epidemiol. 2012 Apr;36(2):222-9. doi: 10.1016/j.canep.2011.06.001. Epub 2011 Aug 15. PubMed 21840283

[Study Results] Yang JL, Lin JJ. Reliability of function-related tests in patients with shoulder pathologies. J Orthop Sports Phys Ther. 2006 Aug;36(8):572-6. PubMed 16915978

[Study Results] Bellet RN, Adams L, Morris NR. The 6-minute walk test in outpatient cardiac rehabilitation: validity, reliability and responsiveness--a systematic review. Physiotherapy. 2012 Dec;98(4):277-86. doi: 10.1016/j.physio.2011.11.003. Epub 2012 May 16. Review. PubMed 23122432

[Study Results] Schmidt K, Vogt L, Thiel C, Jäger E, Banzer W. Validity of the six-minute walk test in cancer patients. Int J Sports Med. 2013 Jul;34(7):631-6. doi: 10.1055/s-0032-1323746. Epub 2013 Feb 26. PubMed 23444095

[Study Results] Ch'ng S, Oates J, Gao K, Foo K, Davies S, Brunner M, Clark JR. Prospective quality of life assessment between treatment groups for oral cavity squamous cell carcinoma. Head Neck. 2014 Jun;36(6):834-40. doi: 10.1002/hed.23387. Epub 2013 Sep 2. PubMed 23720248

[Study Results] Canis M, Weiss BG, Ihler F, Hummers-Pradier E, Matthias C, Wolff HA. Quality of life in patients after resection of pT3 lateral tongue carcinoma: Microvascular reconstruction versus primary closure. Head Neck. 2016 Jan;38(1):89-94. doi: 10.1002/hed.23862. Epub 2015 Jun 16. PubMed 25224592

[Study Results] Bjordal K, Hammerlid E, Ahlner-Elmqvist M, de Graeff A, Boysen M, Evensen JF, Biörklund A, de Leeuw JR, Fayers PM, Jannert M, Westin T, Kaasa S. Quality of life in head and neck cancer patients: validation of the European Organization for Research and Treatment of Cancer Quality of Life Questionnaire-H&N35. J Clin Oncol. 1999 Mar;17(3):1008-19. PubMed 10071296

[Study Results] Chie WC, Hong RL, Lai CC, Ting LL, Hsu MM. Quality of life in patients of nasopharyngeal carcinoma: validation of the Taiwan Chinese version of the EORTC QLQ-C30 and the EORTC QLQ-H&N35. Qual Life Res. 2003 Feb;12(1):93-8. PubMed 12625522

[Study Results] Sherman AC, Simonton S, Adams DC, Vural E, Owens B, Hanna E. Assessing quality of life in patients with head and neck cancer: cross-validation of the European Organization for Research and Treatment of Cancer (EORTC) Quality of Life Head and Neck module (QLQ-H&N35). Arch Otolaryngol Head Neck Surg. 2000 Apr;126(4):459-67. PubMed 10772298

Links:

Available IPD/Information:
